# *MIR2-Toolkit*: a Python program for multiple-image radiography analysis with integrated silicon-crystal diffraction modeling tools

**DOI:** 10.1107/S1600577526008696

**Published:** 2026-09-14

**Authors:** Farangis Foroughi, David Krapohl, Börje Norlin, Dean Chapman

**Affiliations:** a Department of Physics and Engineering Physics, College of Arts and Science University of Saskatchewan 116 Science Place Saskatoon SaskatchewanS7N 5E2 Canada; b Department of Computer and Electrical Engineering Mid Sweden University Sundsvall Sweden; c Department of Anatomy, Physiology and Pharmacology University of Saskatchewan Saskatoon SaskatchewanS7N5E5 Canada; Australian Synchrotron, Australia

**Keywords:** MIR2, multiple-image radiography, analyzer-based imaging, X-ray phase-contrast imaging, dynamical diffraction, silicon crystal

## Abstract

Analyzer-based multiple-image radiography (MIR) is an X-ray phase-contrast imaging technique. It retrieves absorption, refraction, and ultra-small-angle X-ray scattering signals using the angular selectivity of a perfect-crystal analyzer. Conventional MIR processing typically relies on normalization between the object and reference datasets and on precise angular alignment, thereby increasing sensitivity to mechanical and thermal instabilities. The recently developed MIR2 framework addresses these limitations by independently analyzing object and reference datasets, eliminating explicit normalization and global alignment steps, and incorporating angular calibration based on the dynamical theory of diffraction. To facilitate the practical application of this method, *MIR2-Toolkit* has been developed as a Python-based graphical software package for MIR2 analysis. The software carries out dark correction, region-of-interest selection, rocking-curve handling, independent angular calibration, and pixel-wise Gaussian fitting of angular intensity profiles to retrieve contrast images. In addition to the main MIR2 workflow, the toolkit includes silicon-crystal diffraction modules for calculation and visualization of diffraction properties, reflectivity curves, and Darwin widths in symmetric Bragg and Laue geometries. In this paper, the MIR2 workflow implemented in the software is described, the program structure is outlined, and the main analysis and diffraction-modeling features are presented.

## Abstract 2

*MIR2-Toolkit* is an open-source Python program for analyzer-based multiple-image radiography analysis with integrated silicon-crystal diffraction modeling tools and a sample dataset for reproducible evaluation.

## 1. Introduction

Analyzer-based X-ray phase-contrast imaging extends conventional radiography by exploiting the angular selectivity of perfect-crystal diffraction to separate multiple contrast mechanisms from the beam diffracted by an analyzer crystal (Chapman *et al.*, 1997 ▸). In multiple-image radiography (MIR), a sequence of projection images is acquired while the analyzer is stepped through angular positions across its rocking curve (Oltulu *et al.*, 2003 ▸; Pagot *et al.*, 2003 ▸). From these angularly resolved intensity measurements, absorption, refraction, and ultra-small-angle X-ray scattering (USAXS) signals can be retrieved (Diemoz *et al.*, 2010 ▸; Zhang *et al.*, 2018 ▸).

Although MIR has been experimentally demonstrated and applied in various synchrotron settings, practical implementation of the analysis pipeline remains technically demanding. Conventional approaches often rely on explicit normalization between object and reference datasets and assume stable angular alignment throughout acquisition (Wernick *et al.*, 2003 ▸). In realistic beamline environments, however, rocking-curve drift, thermal instabilities, and irregular frame acquisition can occur. These effects complicate quantitative contrast retrieval and reduce reproducibility (Zhang *et al.*, 2007 ▸).

Earlier MIR and DEI studies established that absorption, refraction, and scattering or extinction information can be extracted from pixel-wise analyzer rocking curves by analyzing changes in the peak position, width, and integrated intensity of the curve (Oltulu *et al.*, 2003 ▸; Kitchen *et al.*, 2010 ▸). The MIR2 methodology was recently introduced as a practical reformulation of this workflow, with emphasis on reducing the dependence on direct object/reference image normalization and matched analyzer-angle positions (Foroughi *et al.*, 2025 ▸). In MIR2, after dark correction, the angular intensity profiles at each detector pixel are fitted independently for the reference and object stacks. The MIR contrast channels are then calculated from differences or ratios of the fitted Gaussian parameters, rather than by first normalizing each object image with a corresponding reference image acquired at the same analyzer-angle position. This parameter-based comparison removes the requirement that the object and reference scans be sampled on identical angular grids and improves tolerance to rocking-curve drift, alignment errors, and acquisition irregularities. Angular calibration is performed through a model-based approach grounded in the dynamical theory of diffraction (DTD) (Zachariasen, 1945 ▸; Als-Nielsen & McMorrow, 2011 ▸), allowing discrete acquisition positions or motor units to be expressed in microradian angular units. Although analyzer-based imaging (MIR/DEI) signal-retrieval methods are well established, a documented, open-source, user-accessible software implementation that automates the complete workflow, including angular calibration, pixel-wise fitting, contrast retrieval, visualization, and DTD-based modeling tools, has not previously been available.

In practical synchrotron implementations, analyzer-based imaging additionally benefits from physically consistent diffraction modeling to support angular calibration and interpretation of measured rocking curves. Silicon crystals are widely used for both monochromator and analyzer optics, including at operational beamlines such as the Canadian Light Source BMIT beamline, where Si double-crystal monochromators and Si analyzer crystals are employed. Reproducible MIR2 analysis therefore benefits from an integrated silicon perfect-crystal diffraction module capable of generating physically consistent rocking-curve models.

To address these needs, the *MIR2-Toolkit* has been developed as a Python-based graphical application with a user interface implemented using Streamlit (Streamlit documentation, 2026 ▸). The software provides an end-to-end implementation of the MIR2 analysis pipeline and includes optional silicon diffraction modules to support angular calibration and experiment planning. This article outlines the program structure and presents the main analysis and diffraction-modeling features of the *MIR2-Toolkit*.

## 2. Theory of analyzer-based imaging

Analyzer-based X-ray phase-contrast imaging relies on the angular selectivity of perfect-crystal diffraction. A monochromatic beam, prepared using a double-crystal monochromator (DCM), is diffracted by an analyzer crystal positioned downstream of the sample. The analyzer selects only those beam components whose propagation direction lies within a narrow angular acceptance, typically a few microradians, around the Bragg angle. The angular response of the analyzer system is described by its rocking curve, *R*(θ), which characterizes reflectivity as a function of angular deviation from the Bragg condition.

When a sample is introduced into the beam, three principal modifications of the angular spectrum occur. Absorption reduces the overall transmitted intensity, refraction shifts the angular centroid of the distribution, and USAXS broadens the angular spread. In an MIR experiment, a sequence of projection images *I*(θ_*i*_) is acquired while the analyzer is tilted across the rocking curve. The measured intensity at each pixel therefore samples the angular distribution of the transmitted beam filtered by the analyzer response. To quantify these contrast mechanisms, the angular intensity distribution measured at each pixel is parameterized by a Gaussian model.

### 2.1. Gaussian parameterization, angular calibration, and MIR2 contrast retrieval

Although functions such as Pearson VII and pseudo-Voigt profiles have been proposed to better represent experimental rocking-curve shapes (Kitchen *et al.*, 2010 ▸), Gaussian fitting was selected in MIR2 because it provides robust estimation of the parameters required for MIR analysis (peak position, width, and integrated area) while maintaining a simple and computationally efficient analysis framework. Under the assumption that cumulative small-angle scattering gives rise to a Gaussian angular distribution (Hemmer & Farquhar, 1968 ▸), the transmitted intensity profile at each pixel is modeled as 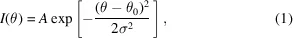
where *A* represents the peak amplitude, θ_0_ denotes the angular centroid, and σ characterizes the angular width.

In the MIR2 framework, the angular calibration procedure was introduced to provide an absolute angular scale for the measured rocking curves. By matching the measured reference rocking curve to a theoretically calculated rocking curve, the calibration converts image indices into angular units without requiring direct microradian-scale measurement of the analyzer motion. The angular calibration is performed independently for the reference and object datasets. A theoretical rocking curve is generated from the dynamical theory of diffraction, and a Gaussian is fitted to this reference curve to determine the corresponding theoretical width σ_th_. To construct the angular scale, a sequence of projection indices, *k*, is used, equally spaced and centered around zero. The angular scale is then calculated by scaling and shifting the projection indices to match the theoretical rocking curve, 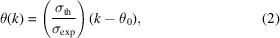
where σ_th_ is the width of the simulated rocking curve, and σ_exp_ and θ_0_ are the width and center obtained from the experimental data in the calibration region. Refracting or scattering structures in the sample can broaden or shift the measured angular profile and bias the calibration. This converts the image-index axis of the measured stack into angular units. In this way, the fitted centroid and width parameters are expressed in physically meaningful angular units for both the reference and object datasets. The calibration region used to estimate σ_exp_ and θ_0_ should be selected from a flat, unstructured part of the image, ideally containing only the beam or a uniformly weakly absorbing material.

After angular calibration, the Gaussian parameters (*A*_ref_, θ_0,ref_, σ_ref_) and (*A*_obj_, θ_0,obj_, σ_obj_) are obtained independently for each pixel in the reference and object image stacks. Quantitative contrast channels are then derived from these fitted parameters.

A shift in the angular centroid relative to the reference condition corresponds to refraction, 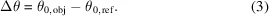
USAXS is associated with the broadening of the angular distribution and is retrieved from the difference in fitted widths as 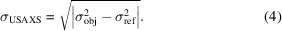
The integrated Gaussian area is proportional to *A*σ, so transmission is computed as 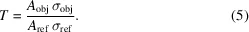
The corresponding radiographic image is then obtained as 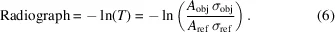
Because fitting is performed independently for the reference and object datasets, no explicit pixel-wise normalization of the object images by the reference images is required before analysis. This Gaussian parameterization forms the basis of MIR2 retrieval by linking sample-induced modifications of the angular spectrum to the corresponding physical contrast mechanisms.

The theoretical framework outlined above defines the MIR2 retrieval procedure implemented in the software. The following sections describe how this workflow is organized computationally within the *MIR2-Toolkit* and how it is presented through the graphical user interface.

## 3. Software architecture, dependencies, and implementation

The *MIR2-Toolkit* is developed in Python as an interactive scientific application with a graphical user interface. The interface is developed using the Streamlit framework, enabling the software to run in a local web browser. Streamlit provides tools for file input, parameter selection, and visualization directly from Python scripts. In the present implementation, Streamlit manages the user interface and workflow logic, while numerical computation is performed in the backend using standard scientific Python libraries.

**Figure 1:**
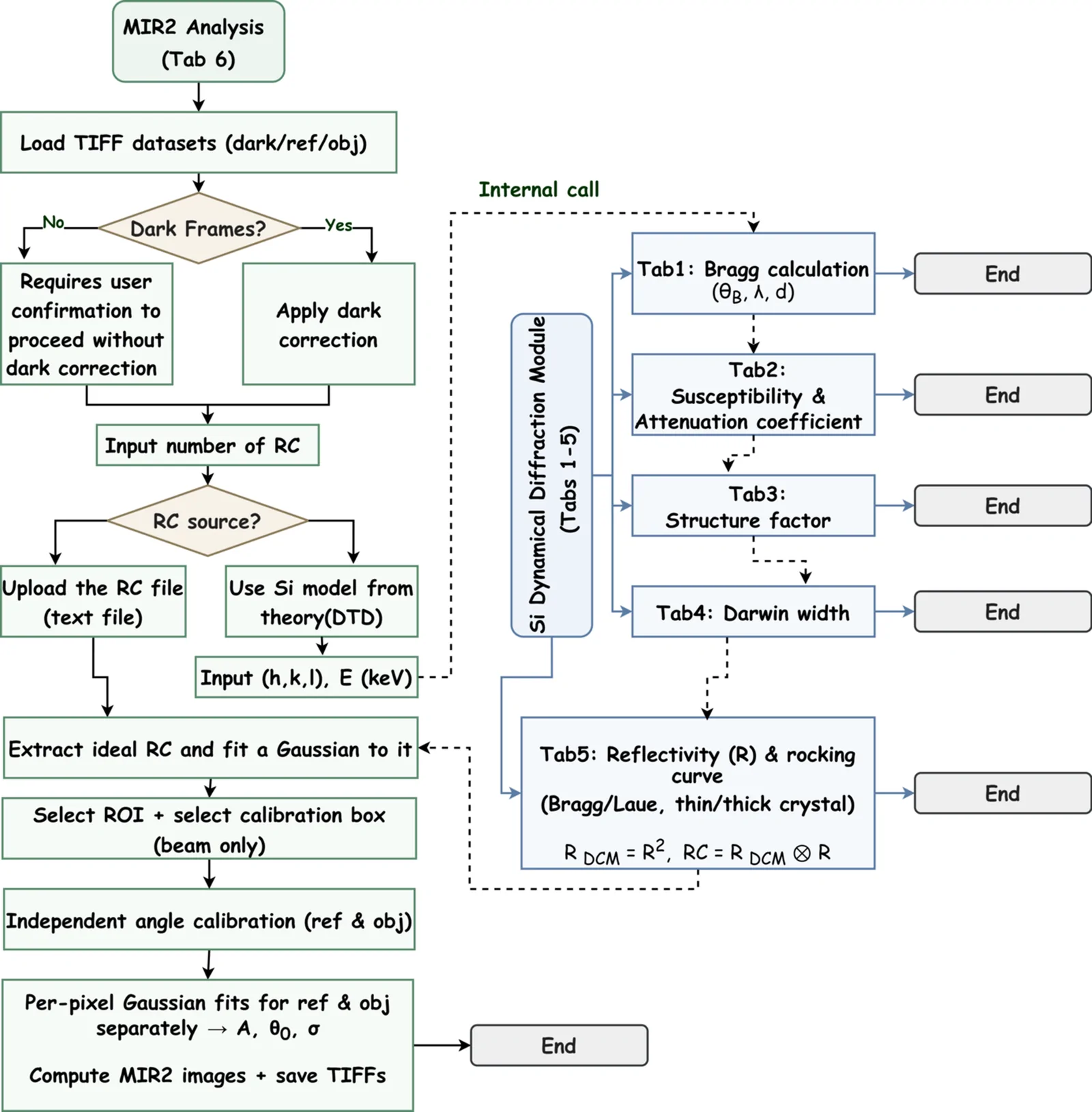
Logical workflow and module organization of the *MIR2-Toolkit*. The main analysis path begins in the MIR2 Analysis tab with loading TIFF datasets, optional dark correction, rocking-curve selection, ROI and calibration-box selection, independent angular calibration of reference and object datasets, and pixel-wise Gaussian fitting to retrieve *A*, θ_0_, and σ. The complementary silicon diffraction module (right branch; tabs 1–5) provides Bragg-angle calculation, susceptibility and attenuation evaluation, structure-factor computation, Darwin-width determination, and reflectivity/rocking-curve modeling. When the silicon model is selected as the rocking-curve source within the MIR2 tab, these calculations are invoked internally, and the user is not required to navigate through the diffraction tabs during MIR2 analysis.

The software architecture centers on the MIR2 analysis workflow for experimental image data, which serves as the core component of the *MIR2-Toolkit*. This workflow implements the main stages of MIR2 processing, from image-stack import and rocking-curve handling to angular calibration, pixel-wise Gaussian fitting, and generation of MIR2 contrast images. In addition, the toolkit includes complementary silicon diffraction modules that provide calculations of Bragg angle, susceptibility, structure factor, Darwin width, and reflectivity. These modules can be accessed directly through their own tabs or invoked internally when the MIR2 workflow requires a theoretical silicon rocking curve. The overall logical workflow and interaction between the MIR2 analysis path and the complementary diffraction modules are illustrated in Fig. 1 ▸.

Numerical array operations are performed using *NumPy* (Harris *et al.*, 2020 ▸), while fitting and related scientific computations are performed using *SciPy* (Virtanen *et al.*, 2020 ▸). Image stacks are stored internally as three-dimensional arrays of the form (*N*, *N*_*y*_, *N*_*x*_), where *N* is the number of angular frames and (*N*_*y*_, *N*_*x*_) are the detector-plane dimensions. This representation allows the same processing logic to be applied to both reference and object datasets, independent of the exact number of angular steps in each stack. For a synchrotron dataset of approximately 4000 × 300 pixels acquired at 14 analyzer angles (14, 300, 4000), *MIR2-Toolkit* processes the full dataset in approximately ten minutes on a standard desktop workstation.

A key implementation feature is the separation between interface-level interaction and backend analysis. User actions such as selecting folders, entering reflection indices, defining the crop region of interest (ROI), or choosing a calibration box are handled in the graphical layer, whereas the corresponding computational tasks are handled by dedicated numerical routines. This separation improves maintainability and facilitates future extension of the toolkit.

The current distribution is intended for local execution on standard research computers running Windows, macOS, or Linux. In the present software release, the toolkit has been tested with Python 3.12, and the recommended installation procedure uses a virtual environment together with package installation from the supplied requirements.txt file.

## 4. MIR2 analysis module and graphical interface

The MIR2 analysis module constitutes the core component of the *MIR2-Toolkit* and implements the complete MIR2 workflow within an interactive graphical environment. This module guides the user from data import and rocking-curve specification to angular calibration, pixel-wise fitting, and generation of the final MIR2 contrast images.

This module accepts three categories of image data: dark frames (optional), reference image stacks, and object image stacks. The image data are provided as TIFF files arranged in separate folders for each dataset. The dark dataset is used for detector-offset correction, the reference dataset represents beam images acquired without the sample, and the object dataset contains the sample projections acquired at different analyzer angles.

If dark frames are provided, dark correction is applied either frame-wise, when the number of dark frames matches the dataset, or using a median-averaged dark image. Continuing without dark correction is only appropriate when the detector response is negligible or when dark correction has already been applied by the detector readout or preprocessing pipeline. For this reason, the software requires explicit user confirmation before proceeding without dark correction.

An ideal rocking-curve model must then be specified. Two options are supported: (i) internally generated silicon rocking curves computed from the integrated dynamical-diffraction module, or (ii) user-uploaded rocking-curve data in text format. In the silicon-model case, the user needs to enter the reflection plane (*h*, *k*, *l*) and the experiment energy. The toolkit then computes single-crystal reflectivity and constructs the corresponding double-crystal rocking curve before fitting it with a Gaussian model to obtain the parameters (*A*_RC_, θ_0,RC_, σ_RC_). In the upload case, the provided angular-intensity data are normalized and fitted in the same manner.

After this stage, middle-angle previews of the reference and object stacks are displayed to allow inspection of beam uniformity and sample positioning. The user then defines a rectangular ROI containing the beam footprint. All subsequent computations are restricted to this ROI in order to reduce computational load and avoid fitting background regions.

**Figure 2:**
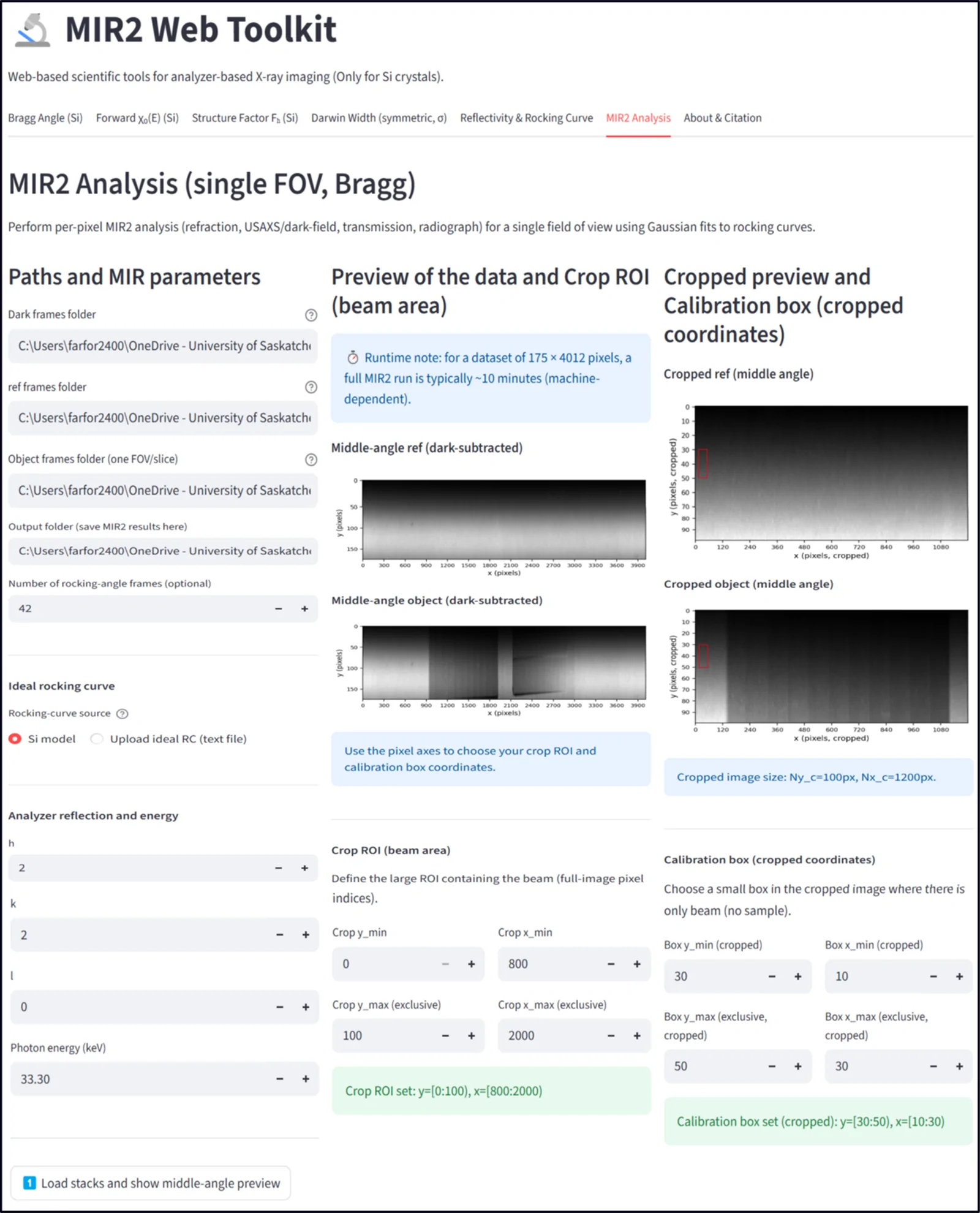
Graphical user interface of the MIR2 analysis module (tab 6). The user specifies dataset paths, rocking-curve source (silicon model or uploaded file), analyzer parameters, and cropping coordinates for the beam-containing ROI. Middle-angle previews of the reference and object stacks assist in selecting the ROI and calibration box. The calibration box must be placed in an unstructured beam-only region to ensure accurate angular calibration.

Within the cropped ROI, a smaller calibration box is selected in a region containing ideally only the beam. The calibration box should be placed in an unstructured region of the image, ideally containing only air or a uniformly low-absorbing material such as thin plastic. Selecting a structured region, such as paper or any heterogeneous sample area, may broaden the measured angular distribution and thereby distort the estimated scattering width. Such bias directly affects the angular calibration and can degrade the quantitative accuracy of the subsequent MIR2 analysis. Fig. 2 ▸ shows the MIR2 graphical interface, where an example of a suitable calibration region is highlighted by a red box within the unstructured area. The summed intensity within the calibration box, as a function of frame index, provides an experimental rocking-curve profile for both reference and object stacks. These one-dimensional profiles are normalized and fitted with Gaussian functions to determine their effective centroids and widths.

Following calibration, pixel-wise Gaussian fitting is performed across the entire cropped ROI. For each pixel, the intensity vector as a function of calibrated angle is fitted using non-linear least-squares optimization (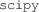.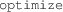.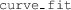). This yields amplitude *A*, centroid θ_0_, and width σ for the reference and object datasets separately.

**Figure 3:**
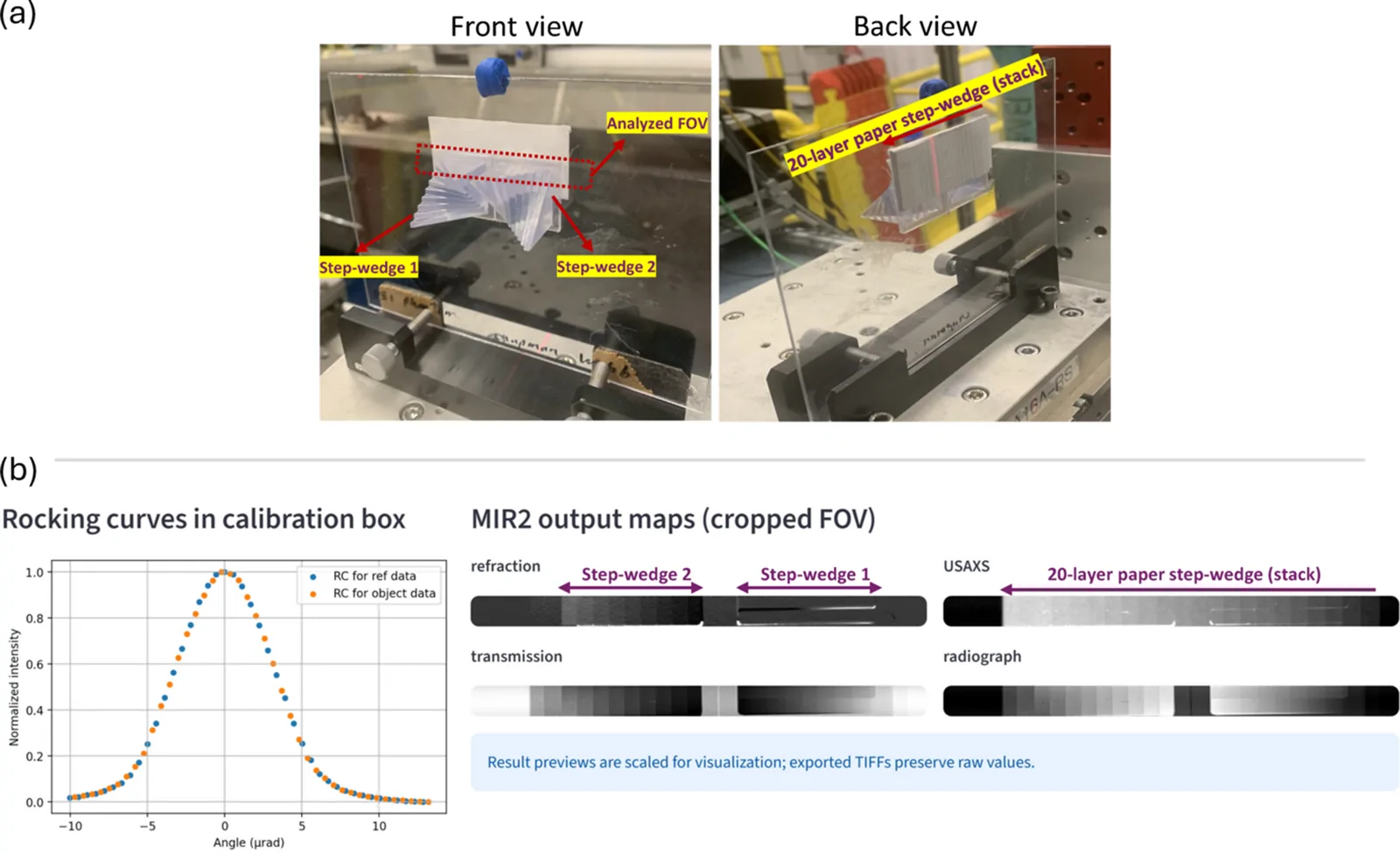
Test object and representative MIR2 results for a single FOV. (*a*) Front and back views of the test object, consisting of two orthogonally oriented PMMA step-wedges (step-wedge 1 and 2) and a 20-layer paper step-wedge (stack). The dashed red box indicates the FOV analyzed in panel (*b*). (*b*) Left: extracted rocking curves from the calibration region for the reference and object datasets after angular calibration. Right: MIR2 contrast maps from the indicated FOV, including refraction, USAXS, transmission, and radiograph. The displayed previews are intensity-scaled for visualization, whereas exported TIFF files preserve the original floating-point values.

Quantitative MIR2 contrast maps are then computed from the fitted parameter maps as described in the previous section. Fig. 3 ▸ shows the test object and representative MIR2 results for a single field of view (FOV) displayed within the graphical interface. The test object consisted of two orthogonally oriented 3D-printed PMMA step-wedges and a 20-layer paper step-wedge (stack), designed to provide attenuation, refraction, and scattering-sensitive features for MIR analysis, as described in the MIR2 methodology paper (Foroughi *et al.*, 2025 ▸). In Fig. 3 ▸(*a*), front and back views of the test object are shown, with the analyzed FOV indicated by the dashed red box. The selected FOV intersects both PMMA step-wedges and the layered paper step-wedge. In Fig. 3 ▸(*b*), the left panel shows the extracted rocking curves for the reference and object datasets after angular calibration. The grayscale images on the right show the corresponding MIR2 contrast maps from the same FOV. The displayed previews are intensity-scaled for visualization purposes, whereas the exported TIFF files preserve the original floating-point values.

## 5. Complementary Si diffraction modules

**Figure 4:**
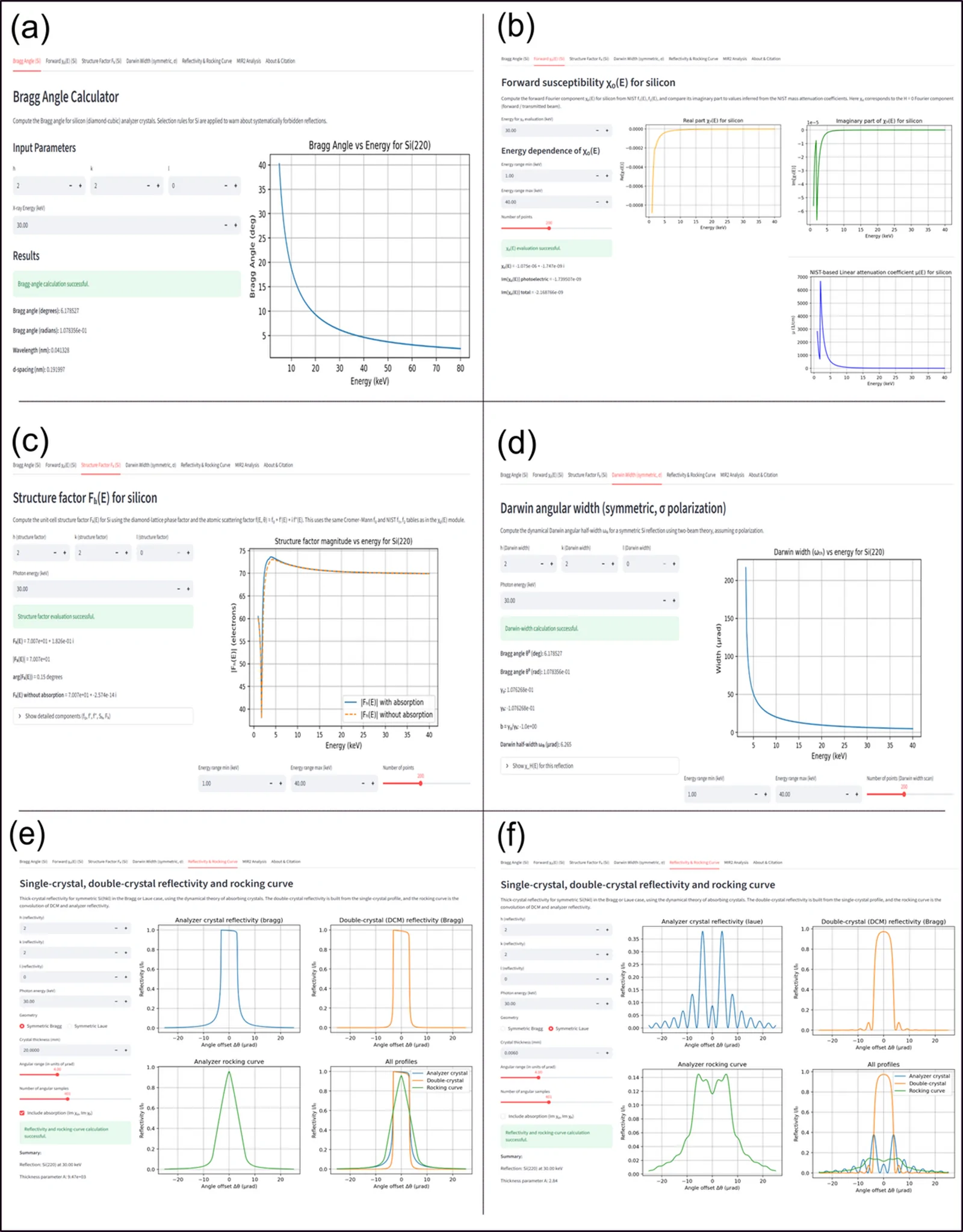
Graphical interface of the complementary silicon diffraction modules (tabs 1–5). The modules provide calculation and visualization of (*a*) Bragg angle, (*b*) forward susceptibility, (*c*) structure factor, (*d*) Darwin width, and reflectivity and rocking-curve response for selected silicon reflections and geometries. Panels (*e*) and (*f*) present reflectivity calculations for symmetric Bragg and symmetric Laue geometries. In panel (*e*), absorption is included; in panel (*f*), the corresponding reflectivity is shown without absorption.

In addition to the core MIR2 analysis module, the *MIR2-Toolkit* includes complementary modules for silicon perfect-crystal diffraction modeling, illustrated in Fig. 4 ▸. These modules provide physically consistent calculation and visualization of Bragg angle, forward susceptibility, structure factor, Darwin width, reflectivity, and rocking-curve profiles. Their role is twofold. First, they allow users to inspect diffraction properties relevant to analyzer-based imaging experiments. Second, when the internal silicon-model rocking curve is selected in the MIR2 analysis workflow, these calculations are invoked automatically in the background to generate the reference rocking-curve model required for angular calibration and Gaussian fitting.

The inclusion of these modules is motivated by the widespread use of silicon crystals in analyzer-based imaging beamlines, including configurations in which both the monochromator and analyzer are based on Si reflections. Integrating these calculations within the same software environment makes the MIR2 workflow more self-contained, reproducible, and transparent.

### 5.1. Bragg angle, susceptibility, and structure factor

The first group of complementary modules provides the Bragg angle and crystal-response quantities required for diffraction modeling. The Bragg-angle module [Fig. 4 ▸(*a*)] computes the diffraction angle for a selected Si reflection and X-ray energy. The susceptibility module [Fig. 4 ▸(*b*)] evaluates the forward electric susceptibility of silicon, while the structure-factor module [Fig. 4 ▸(*c*)] calculates the complex structure factor for the selected reflection. Together, these quantities define the material-specific response of the crystal and provide the basis for subsequent calculation of Darwin width and reflectivity.

These modules are useful both for educational purposes and for practical experiment planning. They allow the user to verify the validity of selected reflections, inspect energy dependence, and better understand how crystal properties influence analyzer acceptance and rocking-curve width.

### 5.2. Darwin width and reflectivity modeling

The Darwin-width module [Fig. 4 ▸(*d*)] calculates the angular acceptance width for the selected reflection and geometry. In the current implementation, the software supports symmetric Bragg and symmetric Laue geometries for the silicon diffraction calculations. The reflectivity module [Figs. 4 ▸(*e*) and 4(*f*)] then uses these quantities to generate single-crystal reflectivity curves and the corresponding double-crystal rocking-curve response used in analyzer-based imaging.

When the silicon-model option is selected inside the MIR2 analysis tab, the toolkit computes the theoretical rocking curve from the specified reflection and X-ray energy, then fits the resulting curve with a Gaussian model to obtain the effective rocking-curve parameters used by the MIR2 pipeline. In this way, the diffraction modules are not isolated utilities, but part of the computational chain that supports MIR2 calibration.

### 5.3. Role within the toolkit

Although the MIR2 analysis tab constitutes the main user-facing workflow, the silicon diffraction modules extend the scope of the software beyond image processing alone. They allow users to examine analyzer behavior independently, compare Bragg and Laue responses, and explore how reflection choice and beam energy affect angular acceptance. This is useful during experiment preparation, method development, and interpretation of measured rocking curves.

The toolkit also supports the upload of an external rocking curve when the analyzer is not silicon, when a measured rocking curve is preferred, or when a custom analyzer model is required. This preserves generality while keeping the silicon case physically grounded and directly integrated with the MIR2 workflow.

## 6. Output products and availability

### 6.1. Output products

The toolkit produces both intermediate preview outputs and final analysis results. During setup, the graphical interface displays a middle-angle preview of the object image together with a preview of the cropped ROI. These previews allow the user to verify sample placement, beam coverage, and the selected analysis region before running the retrieval.

After processing, the software displays the analysis results. These consist of the fitted rocking curves for the reference and object datasets, together with the four retrieved contrast images: the refraction image, USAXS image, transmission image, and radiographic image.

### 6.2. Software availability

The *MIR2-Toolkit* is distributed as an open-source Python program through the public repository *MIR2-Web-Toolkit* (Foroughi & Krapohl, 2026 ▸). The repository includes the application source code, installation instructions, and example datasets that allow users to verify the installation and explore the workflow before applying the toolkit to their own measurements. The current release is intended for research use in analyzer-based X-ray phase-contrast imaging.

## 7. Discussion and conclusion

The *MIR2-Toolkit* was developed to provide a practical and reproducible implementation of the MIR2 framework in a form that can be used directly by experimental researchers. Its main contribution is not the introduction of a new retrieval theory beyond MIR2 itself but rather the integration of MIR2 methodology into a usable software environment that combines image handling, angular calibration, pixel-wise fitting, and physically motivated rocking-curve support within a single interface.

The current version is primarily intended for analyzer-based imaging experiments in which angularly resolved projection stacks are available and Gaussian parameterization of the transmitted angular distribution is an appropriate model. The software is particularly well suited to synchrotron implementations of MIR and related analyzer-based phase-contrast imaging workflows. Because the rocking curve can also be provided externally, the toolkit is not restricted to silicon-only use cases, although the internally modeled diffraction calculations are currently tailored to silicon.

Several limitations should also be noted. First, the quality of the final calibration depends on the appropriate selection of the calibration box; if this region contains structural heterogeneity, the estimated angular width may be biased. Second, the internal diffraction calculations currently focus on symmetric silicon geometries, so other analyzer materials or more general crystal configurations require an external rocking curve. In addition, the present implementation is based on Gaussian fitting of angular intensity distributions, which is appropriate for many MIR applications but may not capture more complex scattering behavior across all systems.

Despite these limitations, the software substantially lowers the barrier to applying MIR2 in practice. Overall, the *MIR2-Toolkit* offers a practical open-source implementation of MIR2 for analyzer-based X-ray phase-contrast imaging. Together with the included example data and documentation, it is intended to facilitate broader use of MIR2 and to support further development of robust multi-contrast imaging workflows.
